# Back to School: Parental Concerns of Children with Hematological and Oncological Conditions During the COVID-19 Pandemic

**DOI:** 10.5334/cie.92

**Published:** 2023-10-02

**Authors:** Katie DiCola, Alexandra Antosy, Dara M. Steinberg

**Affiliations:** 1 Columbia University Medical Center, US

**Keywords:** pediatric, hematology, oncology, education, special education, COVID-19

## Abstract

The Coronavirus Disease 2019 (COVID-19) pandemic has significantly affected the educational system. Historically, children with hematological and oncological conditions have experienced academic challenges. A retrospective chart review was conducted. Charts reviewed included children with oncology or hematology conditions, whose parents expressed educational concerns and were referred to an educational liaison in the Pediatric Hematology, Oncology, and Stem Cell Transplantation Division. The parental concerns for 102 children (*M* age = 10.03 ± 4.7; 59.8% male; 43.1% Latinx/Hispanic) during the first half of the 2021 to 2022 academic year were extracted. Overall, all parents reported at least one concern with the average reporting 2.24 ± 1.34 (range of 1–6 concerns). The most common general concerns regarded: Individualized Education Programs (IEP) or special education evaluations; 504 accommodations; home instruction; school enrollment. Almost half of the parents had additional concerns they specifically attributed to COVID-19. Children with hematological and oncological conditions were experiencing various needs at this time, which likely have continued implications. It is important for educators, school psychologists, and health care providers to remain cognizant of the educational needs of medically vulnerable populations. Children with hematological and oncological conditions benefit from regular evaluation of their needs, as well as proactive forms of intervention.

## Introduction

The Coronavirus Disease 2019 (COVID-19) pandemic has resulted in the most significant disruption in education in history, affecting nearly 1.6 billion children worldwide (UNESCO, 2021). This vast public health crisis led to circumstances that education systems were not prepared for, including mandated social distancing practices and statewide lockdowns. In April 2020, 188 countries, including the United States (US), closed brick-and-mortar schools and explored methods for providing remote instruction (UNICEF, 2020). While school closures related to public health crises have happened in the past, they have never been experienced to this scale (Meyers & Thomasson, 2021). In New York City (NYC), the largest school district in the US and where this study is based, public schools had revolving in-person and virtual learning programs for over a year (Mirakhur et al., 2022).

Similar to other public health crises such as the polio epidemic of 1916 (Meyers & Thomasson, 2021), there is growing evidence that the COVID-19 pandemic may have immediate and long-term academic implications for pre-primary and school-aged children (Chaturvedi et al., 2021; Ford et al., 2021; Minhas & Freeman, 2021). Recent studies have indicated the potential loss of knowledge and academic skills due to virtual learning (Dong et al., 2020; Hasan & Bao, 2020). Additionally, there is growing evidence that virtual learning may have been particularly problematic for young children and other vulnerable populations (Aishworiya & Kang, 2021; Ford et al., 2021; Minhas & Freeman, 2021). Returning to school in-person was also an uncertain time for children, especially for those with medical complexities (Dooley et al., 2020). Many children with complex medical conditions could not be safely vaccinated for COVID-19 or were at increased risk for infection (Sherby et al., 2022).

Children with hematological and oncological conditions (herein referred to as children with hematological/oncological conditions) historically have experienced disruptions in education and lower levels of overall academic achievement compared to healthy peers (Buizer et al., 2006; Devine et al., 2022; Vetsch et al., 2018). Lengthy hospital admissions and frequent medical appointments often result in extensive school absences (Tadmor et al., 2012). Medical treatments and side effects may suppress their immune systems and require them to receive homebound or hospital-based education (Adduci et al., 2021). These services promote school engagement and provide a sense of normalcy throughout treatment (Adduci et al., 2021; Eiser & Vance, 2002). Their limitations include fewer hours of instruction, lack of peer interactions, and less access to special education services (Ahumada-Newhart & Olson, 2019). Medical treatments such as intrathecal chemotherapy and radiation at a young age put children at risk for developing neurocognitive deficits including but not limited to challenges with executive functioning, fine motor dexterity, memory, processing speed, and diminished Intelligence Quotient (IQ) (Children’s Oncology Group, 2018; Roddy & Mueller, 2016).

Returning to school after medical treatment can be a challenging experience for children with hematological/oncological conditions (Klein et al., 2022). These challenges also extend to school staff who may feel unprepared if they do not have sufficient information or training to address the student’s physical, psychosocial, and academic needs (Klein et al., 2022). There are several existing school re-entry programs that have successfully addressed the psychosocial needs of this population, but there has been limited research on programs that include academic support (Helms et al., 2016; Thompson et al., 2015). Federal Education laws and regulations within the US such as the Individuals with Disabilities Act (IDEA) and the Section 504 of the Rehabilitation Act mandate academic accommodations and provide protection for students with hematological/oncological conditions (United States Department of Education, 2023).

Under Section 504 of the Rehabilitation Act, students with hematological/oncological conditions qualify for a 504 Plan which provides students with medical conditions accommodations that facilitate access to school programs and activities (United States Department of Education, 2023). These accommodations may include testing accommodations (e.g., extended time on examinations and isolated testing location), classroom accommodations (e.g., excused absences, flexible class schedule and repeated instructions), and academic support (e.g., paraprofessional and nursing services) (New York City Department of Education, 2023). While these accommodations are academic based, they do not include modifications of course content and are intended to provide students with disabilities an equal opportunity to access resources and activities in a general education classroom (Deutch et al., 2015). 504 Plans are commonly used during school re-entry for medically complex children who are not in need of intensive academic support (Hopkins & Hughes, 2016).

The Individuals with Disabilities Act (IDEA) entitles all students to a Free and Appropriate Education (FAPE) that requires all public schools to provide special education services to qualifying students (United States Department of Education, 2020). To qualify for special education services, students must fall within at least one of the 13 disability classifications (e.g., Other Health Impairment) under FAPE and the disability must impact the student’s ability to learn (The University of the State of New York, 2002). Qualifying students may be eligible for a classroom setting outside general education (i.e., Integrated Co-Teaching or Special Education class), resource room (i.e., pull-out services), consultant teacher services (i.e., push-in services), and related services (e.g., speech therapy, occupational therapy, and physical therapy) through an Individualized Education Program (IEP) (New York State Education Department, 2013). Research has shown that medically complex students, such as children with hematological/oncological conditions are more likely to require special education services than their health peers (Mitby, 2003; Norman, 2016). There is also evidence to suggest that special education services can prevent declines in neurocognitive functioning (Butler, 2008).

Children with hematological/oncological conditions may be at further risk due to the educational disruptions of the COVID-19 pandemic. The consequences of COVID-19 on education are continuing to emerge, thus research on these children is limited. To begin to understand their experiences and potential current as well as future needs, parental educational concerns (herein referred to as concerns) during the first half of the 2021–2022 school year (approximately 1.5 years into the COVID-19 pandemic in the US) were evaluated. This study sought to identify: (1) the most common general educational concerns; (2) the most common concerns parents identified as specifically related to COVID-19 (herein referred to as COVID-19 specific concerns); (3) the most common concerns for children with an IEP. It was hypothesized that the most common general concerns and concerns within the group of children with an IEP would be difficulties accessing educational accommodations. It was hypothesized that the most common COVID-19 specific concerns would be participating in virtual learning and return to school with risk of infection and COVID-19 policies. A secondary analysis was done to examine if acculturation (using language as a proxy) was related to general and COVID-19 specific concerns. It was hypothesized that those who spoke a primary language other than English would have greater rates of all concerns. This hypothesis was based on research which has highlighted the role that language proficiency, immigration status, socioeconomic status, race, and ethnicity have on interactions with the school system. Those who are more recent immigrants and are not fluent in English often have children who are more disadvantaged academically (Olivos & Mendoza, 2010).

## Methods

### Data Extraction

The Institutional Review Board (IRB) of Columbia University Medical Center approved a retrospective chart review of children from the Pediatric Hematology, Oncology and Stem Cell Transplantation Division whose parents had concerns and educational liaison consultation from August 1, 2021 to November 22, 2021. A waiver of consent was granted. This time period was chosen to capture the needs of children as they entered and began the 2021–2022 academic year. One author (AA) manually reviewed the charts of all children who met with the educational liaison during the identified time period and extracted the variables of interest. Unknown information was discussed with the other two authors who further reviewed the medical record to determine if information could be identified.

The educational liaison (KD) served as a connection between the children’s health care teams and schools. She regularly met with parents and their children to address concerns they expressed. The main aim of these meetings was to identify the concerns, and gather related educational information (e.g., grade level, existing academic accommodations, academic performance). Meetings occurred in the language parents preferred using telephonic or video interpretation services when not in English. The liaison helped schools understand the children’s medical diagnoses, treatment plans, and anticipated educational needs based on their diagnoses, treatment and related sequelae. This service was available for all children within the division.

### Participants

Charts of children who met the following eligibility criteria were included: educational liaison was consulted by a concerned parent or provider; the child had a diagnosis for which he/she was followed by the Pediatric Hematology, Oncology and Stem Cell Transplantation Division; the child was between ages 2 to 18. This age range was chosen to capture children eligible for school-based services through the US public school system. Although children may be eligible through age 21, no individuals met the criterion.

### Sociodemographic and Background Information

The following information about the child was extracted from the medical record: age at time of educational liaison consultation; biological sex; primary medical condition for which they were treated in the division; time since original medical diagnosis (in years); grade for the 2021–2022 academic year; academic setting; whether they had academic accommodations prior to medical diagnosis; type of insurance (i.e., public or private). Parent’s primary language was extracted as a proxy for acculturation (Miranda et al., 2011).

### General and COVID-19 Specific Concerns

Parent-reported concerns were extracted from the educational liaison notes within the medical record. All concerns were extracted thus multiple concerns from a single parent were included. They were then categorized and coded. Those concerns parents specified due to the COVID-19 pandemic, were analyzed separately. Children with special education services or in the process of being evaluated for them (in this case defined as having an IEP or being evaluated for an IEP) were included in the main analyses, and then further examined as a separate group.

### Statistical Analysis

Sociodemographic and background information were analyzed using measures of central tendency (i.e., frequencies, means, standard deviations). General concerns, COVID-19 specific concerns, and concerns specifically for children with special educational services were also analyzed using measures of central tendency. A secondary analysis was conducted to determine the relationship between acculturation (using parental primary language as a proxy) and frequency of general and COVID-19 specific concerns. Multiple chi-square analyses were done comparing English-speaking and non-English speaking parents and whether or not they had each of the 24 overall concerns identified. Statistics were done using SPSS Version 26 and a significance level of *p* < .05 was set.

## Results

### Participants

#### Sociodemographic Background

The parents of 102 children between the ages of 2 to 18 years old (*M* age = 10.03 ± 4.7) had educational concerns. The children were 59.8% male (*n* = 61) and 43.1% (*n* = 44) Latinx/Hispanic. The majority (*n* = 68, 66.7%) had public insurance. Over one-third (*n* = 37, 36.3%) of their parents spoke a language other than English (Table 1). There was a significant relationship between parental primary language and ethnicity as well as type of insurance. Parents whose primary language was not English were more likely to have children who were identified as Hispanic/Latinx (*X^2^* (1, *N* = 95) = 17.44, *p* < .001) and whose insurance was public (*X^2^* (1, *N* = 100) = 14.48, *p* < .001). There was no relationship found between parental primary language and frequency of the 24 general and COVID-19 specific concerns.

**Table 1:** Sociodemographic & Academic Characteristics of the Children (N = 102).


VARIABLE	*n*	%

***Biological Sex***		

Male	61	59.8

Female	41	40.2

Age, *M* ± SD	10.03 ± 4.7	

***Ethnicity***		

Non-Latinx/Hispanic	51	50.0

Latinx/Hispanic	44	43.1

Unknown	7	6.9

***Insurance***		

Public	68	66.7

Private	32	31.4

Unknown	2	2.0

***Parent’s Primary Language***		

English	65	63.7

Spanish	30	29.4

Other	7	6.9

***Hematological/Oncological Condition***		

Leukemia/Lymphoma	31	30.4

Post-Bone Marrow Transplant	22	21.6

Brain Tumor	21	20.6

Solid Tumor	15	14.7

Non-malignant Hematology	8	7.8

Non-malignant Tumor	3	2.9

Unknown	2	2.0

Years since diagnosis, *M* ± SD	4.22 ± 4.6	

**Academic Information**		

***Grade –2021-2022***		

Early Intervention	1	9.8

Pre-K	15	14.7

Kindergarten	6	5.9

Elementary School	31	30.4

Middle School	10	9.8

High School	28	27.5

College	2	2.0

Specialized Day Program (non-degree)	4	3.9

Unknown	5	4.9

***Educational Modality 2020-2021***		

In-person	15	14.7

Virtual	19	18.6

Homebound	9	8.8

Under 5 & not enrolled	15	14.7

Unknown	44	43.1

***Educational Modality 2021-2022***		

In-person	58	56.8

Virtual	7	6.9

Hybrid	2	2.0

Homebound	15	14.7

Under 5 & not enrolled	6	5.9

Not enrolled	2	2.0

In person & then homebound	2	2.0

Unknown	10	9.8

***Accommodations 2020-2021***		

None	50	49.0

IEP	29	28.4

504 Accommodations	10	9.8

Early Intervention	8	7.8

Other related services through insurance	2	2.0

Other	1	1.0

Unknown	2	2.0


#### Medical Background

Children had a range of hematological/oncological diagnoses. The most common diagnoses were liquid tumors (i.e., leukemias and lymphomas; *n* = 31, 30.4%) and children post-stem cell transplantation for malignant and non-malignant conditions (*n* = 22, 21.6%). Time since diagnosis was variable (*M* = 4.22 ± 4.6 years). Data on the stage of treatment children were in was not extracted (Table 1).

#### Educational Background

Most of the children were either in elementary school (*n* = 31; 30.7%) or high school (*n* = 28; 27.7%). Almost half did not have any academic accommodations prior to their medical diagnosis (*n* = 50; 49.5%). During the previous school year (2020–2021) methods of instruction included virtual (*n* = 19; 18.6%), in-person (*n* = 15; 14.7%), and home instruction (*n* = 9; 8.8%). More than half of the children (*n* = 58, 56.9%) were returning/had returned to in-person school during the 2021–2022 school year (Table 1). Due to the nature of the retrospective chart review, the education modality for 44 children was unknown.

#### General and COVID-19 Specific Concerns

On average a total of 2.24 ± 1.34 concerns (general and COVID-19 specific) were reported, with parents reporting a range of 1 to 6 concerns. There was a total of 24 different types of concerns identified.

**General Concerns.** The most common concerns were related to: individualized education programs (IEP) or special education evaluations (*n* = 30, 29.4%); 504 accommodations (*n* = 27, 26.5%); home instruction (*n* = 22, 21.6%); school enrollment (*n* = 20, 19.6%) (Table 2).

**Table 2:** *General Concerns of all parents (N* = 102).


CONCERN	NUMBER OF PARENTS*	PERCENTAGE OF PARENTS

IEP or Special education evaluation	30	29.4

504 accommodations	27	26.5

Home instruction	22	21.6

School placement/enrollment	20	19.6

General referral	11	10.8

Neuropsychological testing	10	9.8

Issues with accommodations	9	8.8

School re-entry	3	3.0

Academic concerns	2	2.0

Early Intervention	1	1.0

Education or legal advocacy	1	1.0

College applications	1	1.0

Non-education need	1	1.0


*Parents could report multiple concerns/needs.

**COVID-19 Specific Concerns.** Although not asked specifically about COVID-19 related concerns, approximately half of parents (*n* = 50, 49%) reported concerns which they identified as specific to COVID-19. Further analysis within this group of parents found that these included: increased risk for infection during school re-entry (*n* = 23, 22.5%); delays in overall services due to limited school district staff (*n* = 16, 15.7%); delays in services listed on the child’s IEP or 504 (*n* = 10, 9.8%) (Table 3). Additionally, several parents (*n* = 18, 17.6%) identified benefits related to COVID-19. These included: the option for virtual home instruction (*n* = 7, 6.9%), hybrid learning (*n* = 4, 3.9%), flexibility with school accommodations and special education evaluations (*n* = 4, 3.9%), and being able to participate virtually in traditional instruction (*n* = 3, 2.9%).

**Table 3:** Concerns of the 50 parents who reported COVID-19 specific concerns.


COVID-19 SPECIFIC CONCERNS	NUMBER OF PARENTS*	PERCENTAGE OF PARENTS

Risk of infection	23	46.0

Delays due to limited staff	16	32.0

Delays in IEP/504 services	10	20.0

Virtual learning or services	9	18.0

Delays in home instruction	8	16.0

Delays in school placement/enrollment	6	12.0

Delays in special education evaluations	6	12.0

Vaccination status	5	10.0

Contracted COVID-19	3	6.0

Mask mandates	2	4.0

Virtual special education evaluations	2	4.0


*Parents could report multiple concerns/needs.

**Children With Special Education Services.** Further analysis of the group of children (*n* = 49, 48%) with an IEP or in the process of being evaluated for one, was done. Delays or interruptions in related services (*n* = 7, 14.2% of those with special education services), and special education evaluations (*n* = 7, 14.2%) were the most prevalent concerns. Challenges with virtual learning were also reported (*n* = 5, 10.2%). There were also challenges in related services including: delays in specialized transportation (*n* = 6, 12.2%); delays in paraprofessional assignment (*n* = 6, 12.2%); challenges with virtual related services (*n* = 5, 10.2%); delays in therapies (e.g., occupational therapy, physical therapy, and speech therapy) (*n* = 5, 10.2%); discontinued related services (*n* = 2, 4.1) (Table 4).

**Table 4:** Parent-reported concerns/needs for the 49 children with an IEP or who requested an IEP evaluation.


CONCERNS	NUMBER OF PARENTS*	PERCENTAGE OF PARENTS

Delays or interruptions in related services	7	14.2

Delays in special education evaluations	7	14.2

Challenges with virtual learning	5	10.2

Delays in IEP amendment meetings	4	8.1

Discontinued virtual learning	2	4.1

Declines in academic performance	1	2.0

Delays in IEP annual review meetings	1	2.0

Delays in special education school placement	1	2.0

***Challenges with related services***		

Delays in specialized transportation	6	12.2

Delays in paraprofessionals	6	12.2

Challenges with virtual related services	5	10.2

Delays in therapies (OT, PT, ST)	5	10.2

Discontinued related services	2	4.1


*Parents could report multiple concerns/needs.

## Discussion

Children with hematological/oncological conditions were experiencing various needs during the first half of the 2021–2022 academic year. Children had a range of medical conditions and were diverse in terms of sociodemographic characteristics. There was no relationship between parental primary language spoken and concerns. This may suggest the pervasiveness of educational needs throughout the population of children with medical conditions.

As hypothesized the most frequently reported general concerns were related to children’s specialized academic needs, specifically IEP evaluations/special education evaluations and 504 accommodations. These findings are consistent with existing research that has shown children with hematological/oncological conditions may require school accommodations such as 504 plans and special education services (Mitby et al., 2003; Moore et al., 2009; Northman et al., 2015; Lorenzi et al., 2009). While these concerns may have pre-dated the pandemic, they continued to be experienced and were likely exacerbated given the level of educational disruption during the pandemic.

Disruption in access to these services may have a cumulative effect, especially given that late effects may emerge anytime from months to years after treatment and may be overlooked or misinterpreted by schools (Mertens & Marchak, 2015; Wakefield et al., 2010). Access to special education services may not prevent late effects but can help these children maintain skills and prevent further cognitive declines (Butler et al., 2008; Moore et al., 2000). It is generally recommended that all these children receive a neuropsychological evaluation or are evaluated by their school district’s special education to identify potential deficits (Hoffman, 2017).

With regard to the COVID-19 pandemic, the current study identified a number of COVID-19 specific concerns. The most common were: risk of infection; delays in implementation of services due to limited staff; delays in establishing IEPs and 504 plans. In March of 2020, the US Department of Education (USDE) announced that all schools must be compliant to IDEA by providing the services listed on every child’s IEP through remote instruction to the extent possible (Jameson et al., 2020). Providing related services (e.g., occupational therapy, physical therapy, speech therapy, and counseling) to children with IEPs was challenging for schools who heavily relied on parent assistance (Dooley et al., 2020; Tremmel et al., 2020). If these services were not provided, the child’s IEP team was required to determine eligibility for compensatory services upon return to in-person learning (Jameson et al., 2020). Consequently, many students with disabilities may not have sufficiently received all mandated services on their IEP during the 2019–2020 school year. Additionally, delays in IEP initial and re-evaluations may have led to children having unrecognized needs for which services would have been beneficial.

It is also important to note that while there were a number of concerns related to the pandemic, there were also some benefits reported. The pandemic led to widespread virtual options for school, home instruction, evaluations, and other services. Prior to universal forms of virtual learning being implemented during the COVID-19 pandemic, alternative strategies such as the use of telepresence robots were used to help address some of these limitations by allowing children to participate in school in real-time. Research has found that telepresence robots have helped students receiving home instruction feel more included but ultimately cannot provide full inclusion in the way that in-person learning can (Ahumada-Newhart & Olson, 2019). Yet, telepresence robots are also not always an accessible option and there is limited research on using them to provide special education services.

The vulnerability of this population is highlighted by the finding that almost half did not have special education services before diagnosis, but had them at the time of data collection. The limitations of virtual learning have mostly been seen in vulnerable populations such as, medically complex children, marginalized children, children under six years of age, and children with learning, developmental or sensory disabilities (Brandenburg et al., 2020; Dayal & Tiko, 2020; Dias et al., 2020; Dreifuss-Serrano & Herrera, 2020; Szente, 2020). Many of these children may also receive special education services which were provided virtually but not necessarily in full during the 2019 to 2020 school year (Jameson et al., 2020).

## Limitations and Future Research

The current findings should be considered in relation to its limitations. As the study was a retrospective chart review, data was gathered and recorded for clinical purposes, thus some information was unknown. Results were based on concerns reported to the education liaison; however, it is possible that if parents were prompted in a more structured manner (e.g., list of potential concerns; asked if they had specific COVID-19 related concerns), they may have responded differently. Additionally, although educational liaison services are available for all children in the division, there may have been differences in which providers thought to refer children and which did not, that biases the data.

Children had a range of diagnoses, illness severities and were at different points of the medical treatment process. The variability of the patient population makes it difficult to conduct between group analyses, but does suggest that the needs of these individuals are somewhat consistent between conditions which is important when considering how to best serve their needs. The children in this study were from diverse backgrounds, yet all were from a similar geographic area. Some of the findings may be unique to challenges and experiences of the school districts in NYC and surrounding areas.

Future research looking at academic performance and outcomes during this time period would be beneficial. In addition, although the sample size was relatively large, it is possible that more children would have been identified if a different time point was chosen. The educational liaison had recently begun her job in August 2021 after a period of time that the position was unfilled. Thus, providers may have had less understanding/awareness of the role affecting referrals. It was also the beginning of the training year for new medical fellows treating these children and they were likely still learning the intricacies of the NYC school system and when to refer. Future research should utilize a longitudinal design to better capture concerns over time both in relation to patient specific variables (e.g., time elapsed since treatment, grade) and general variables (e.g., concerns based on time of year). Research should also evaluate the efficacy of provider-specific trainings on addressing academic needs and concerns for patients.

## Practice Implications

While the impact of the COVID-19 pandemic will likely continue to emerge through years of research, this study highlights important considerations for practice that can be taken at this time.

Immediate action can be taken by school personnel/education systems by implementing preventative interventions to negate the adverse consequences of the COVID-19 pandemic, medical treatment, and school absenteeism experienced by students with hematological/oncological conditions. As previously stated, this population is at risk for neurocognitive deficits that may not be detected until months to years following medical treatment (Children’s Oncology Group, 2018; Roddy & Mueller, 2016). Conducting comprehensive evaluations for special education services such as psycho-educational, behavioral (e.g., executive function and attention), and related services (e.g., speech and language therapy, physical therapy, and occupational therapy) are ideal to determine eligibility for an IEP and provide a thorough understanding of areas of vulnerability. It is possible that these deficits may not be apparent or within the eligibility criteria for services depending on where the student is in their treatment trajectory. As a result, many children with hematological/oncological conditions may not initially qualify for academic interventions that may prevent further academic/neurocognitive decline. Ultimately, schools should not wait until students fail to implement academic interventions.

Prior to and following the COVID-19 pandemic, various school-based prevention programs have been used to support the academic and mental health needs of at-risk students (Capurso et al., 2020; Capurso et al., 2022; Fuchs & Deshler, 2007). Response to Intervention (RTI) is one example of a widely used three-tier intervention in the US that aims to identify and support students who are struggling or at risk for qualifying for special education/specialized instruction (Alahmari, 2019; Fuchs & Deshler, 2007). All students are eligible for Tier 1 which includes using screening methods to identify students who are struggling within general education classrooms. Students can qualify for at-risk services such as small group (Tier 2), additional instruction time (Tier 2), and intensive instruction that includes programs to target the student’s needs (Tier 3) (Alahmari, 2019; New York City Department of Education, 2023). RTI or similar at-risk academic interventions in conjunction with a 504 plan may benefit all children with hematological/oncological conditions who do not meet the criteria for special education services. Additionally, all children with an IEP prior to medical treatment should be re-evaluated prior to school re-entry and receive compensatory services for any gaps in education or related services during the COVID-19 pandemic.

## Conclusion

In summary, as the COVID-19 pandemic continues it will be important for hospital and school-based educators and psychologists to understand and be aware of the unique challenges children with hematological/oncological conditions face surrounding school. While children with hematological/oncological conditions make up a small percentage of all students, their needs are significant, and it is important for those working with them to be educated on the importance of comprehensive assessments and how to act to ensure that appropriate accommodations (504 plan and IEP) and interventions (at-risk services) are implemented upon school re-entry. Educators and School Psychologists should also be aware of pandemic specific concerns and how they may affect children with hematological/oncological conditions short-term and long-term. It is important to remain aware that evolving COVID-19-related school policies may not always align with the best interests of children with hematological/oncological conditions. Schools may want to strive to assess children at multiple points throughout their illness trajectory and make modifications, when necessary, as education needs may change over time.
